# Exploring the current approach to reablement for community‐dwelling people living with dementia: preliminary practitioner‐led goals and targets for practice change

**DOI:** 10.1002/alz.087838

**Published:** 2025-01-09

**Authors:** Claire O'Connor, Christopher Poulos, Susan Kurrle, Kaarin J. Anstey

**Affiliations:** ^1^ School of Psychology, Faculty of Science, University of New South Wales, Sydney, NSW Australia; ^2^ HammondCare, Sydney, NSW Australia; ^3^ Neuroscience Research Australia, Sydney, NSW Australia; ^4^ Ageing Futures Institute, University of New South Wales, Sydney, NSW Australia; ^5^ University of New South Wales, Sydney, NSW Australia; ^6^ Centre for Positive Ageing, HammondCare, Hammondville, Sydney, New South Wales, Australia, Hammondville, NSW Australia; ^7^ University of Sydney, Sydney, NSW Australia; ^8^ UNSW Ageing Futures Institute, Sydney, NSW Australia

## Abstract

People living with dementia have a range of functional, physical, cognitive, and behaviour needs. Reablement (and/or ‘rehabilitation’) is an important multidisciplinary approach, such as using occupational therapy and exercise, to address each person’s needs. People with dementia in Australia are being referred to community aged care teams for everyday living support, and an evidence‐informed reablement handbook is freely available (www.hammond.com.au/reablement). Despite this, evidence‐informed reablement is still not routinely available. This project aims to explore how reablement is currently provided for people with dementia in the community, and to identify practitioner perspectives around potential goals and targets for practice change. The preliminary outcomes from this research (presented here) will feed into a broader project that seeks to understand how to implement evidence‐informed reablement for people with dementia in Australia.

To explore how reablement is currently provided for people with dementia, a retrospective file audit was conducted with two community aged care providers in New South Wales, Australia. At the time of data collection, the providers had not implemented the evidence‐informed reablement handbook into practice. Data from the clinical file notes from each provider were compared against the reablement handbook. Site‐specific outcomes were then presented back to the respective allied health teams from each provider via focus groups; through this process, practitioners identified potential goals and targets for practice change.

Data from 10 clients with cognitive impairment or dementia were included in the audit. There was partial alignment with the handbook in terms of interventions delivered (e.g. personally prescribed multicomponent exercises; provision of compensatory strategies), and some areas where discrepancies existed (e.g. very few written support plans provided to clients). Twelve allied health professionals (occupational therapy, physiotherapy, social work, dietetics) participated in the focus groups. A range of potential targets for practice change were identified, such as better tailored information for clients’ cognitive/physical functioning, and more structured functional cognition assessment.

Although some alignment with the reablement handbook existed, there was room for improvement in provision of reablement for people with dementia. Moving forward, broad consultation with a range of stakeholders is needed to determine the best implementation approach on a national scale.

